# Elevated atmospheric CO_2_ alters the multi-element stoichiometry of pollen-bearing oak flowers, with possible negative effects on bees

**DOI:** 10.1007/s00442-024-05610-2

**Published:** 2024-09-08

**Authors:** Zuzanna M. Filipiak, Carolina Mayoral, Sophie A. Mills, Scott A. L. Hayward, Sami Ullah

**Affiliations:** 1 Institute of Environmental Sciences, Jagiellonian University, Gronostajowa 7, 30-387 Kraków, Poland; 2 Birmingham Institute of Forest Research, University of Birmingham, Edgbaston, Birmingham, B15 2TT England; 3 School of Biosciences, University of Birmingham, Edgbaston, Birmingham, B15 2TT UK; 4 School of Geography, Earth and Environmental Sciences, University of Birmingham, Edgbaston, Birmingham, B15 2TT UK

**Keywords:** FACE, Pollinator, Nutrition, Climate change, Temperate forest, *Quercus robur*

## Abstract

Increasing atmospheric CO_2_ levels change the elemental composition in plants, altering their nutritional quality and affecting consumers and ecosystems. Ecological stoichiometry provides a framework for investigating how CO_2_-driven nutrient dilution in pollen affects bees by linking changes in pollen chemical element proportions to the nutritional needs of bees. We investigated the consequences of five years of Free Air CO_2_ Enrichment (FACE) in a mature oak-dominated temperate forest on the elemental composition of English oak (*Quercus robur*) pollen. We measured the concentrations and proportions of 12 elements (C, N, P, S, K, Na, Ca, Mg, Cu, Zn, Fe, and Mn) in *Q. robur* pollen-bearing flowers collected from the Birmingham Institute for Forest Research (BIFoR) FACE facility. An elevated CO_2_ (eCO_2_) level of 150 ppm above ambient significantly reduced the S, K, and Fe levels and altered the multi-element ratio, with different elements behaving differently. This shift in pollen multi-element composition may have subsequent cascading effects on higher trophic levels. To assess the impact on bees, we calculated the stoichiometric mismatch (a measure of the discrepancy between consumer needs and food quality) for two bee species, *Osmia bicornis* (red mason bee) and *Apis mellifera* (honey bee), that consume oak pollen in nature. We observed stoichiometric mismatches for P and S, in pollen under eCO_2_, which could negatively affect bees. We highlight the need for a comprehensive understanding of the changes in pollen multi-element stoichiometry under eCO_2_, which leads to nutrient limitations under climate change with consequences for bees.

**Supplementary Information:**

The online version contains supplementary material available at 10.1007/s00442-024-05610-2.

## Introduction

Human activities have altered the global chemical element cycles, affecting the biosphere and ultimately compromising human wellbeing (da Silva and Williams 2001; Sterner and Elser 2002) and pollinator health (Jamieson et al. 2017; Wilson and Fox 2021). Anthropogenic greenhouse gas emissions, mainly elevated atmospheric carbon dioxide (eCO_2_), exacerbate these negative effects (Loladze 2014; Ziska et al. 2016; Welti et al. 2020). Additional long-term global changes are expected if greenhouse gas emissions continue at current levels (Calvin et al. 2023).

Increases in atmospheric CO_2_ have been shown to affect various aspects of plant physiology, including but not limited to changes in productivity, intrinsic water-use efficiency, photosynthesis, and plant nutritional quality (Loladze 2014; Ainsworth and Long 2021; Gardner et al. 2022b, 2023). In conjunction with these changes, plant tissues and products exhibit changes in the concentrations and proportions of their constituent chemical elements under eCO_2_, i.e., plant tissues and products undergo changes in stoichiometry (Loladze 2014; Dong et al. 2018). For instance, shifts in multi-element stoichiometry, with different elements displaying varying extents of change, have been observed in the foliage of deciduous and coniferous trees under eCO_2_ (Le Thiec et al. 1995; Gielen et al. 2003; Günthardt-Goerg and Vollenweider 2015), with accompanying effects on foliage physiology (Gielen et al. 2003; Günthardt-Goerg and Vollenweider 2015). Under eCO_2,_ plant biomass increases through increased carbohydrate production, accompanied by changes in plant chemical composition, resulting in decreased concentrations of vital nutrients (Robinson et al. 2012; Welti et al. 2020; Kaspari and Welti 2024). This phenomenon, known as the nutrient dilution hypothesis, results in an increased ratio of carbon to other elements, represented by C:X (where C = carbon and X = other elements), leading to decreased nutritional quality of plant tissues and products (Welti et al. 2020; Kaspari and Welti 2024). Although the greatest shift is anticipated for the C:N (carbon-to-nitrogen) ratio, due to the combined effect of increased carbohydrate and decreased protein levels, decreases are also expected for other elements, such as phosphorus (P), sulphur (S), potassium (K), or iron (Fe) (Loladze 2014). Altered stoichiometry and nutrient dilution due to eCO_2_ have been recognised as global challenges for human health (Loladze 2014). However, increasing concerns also revolve around the negative impacts of these phenomena on other ecosystem components, including herbivores (Robinson et al. 2012; Welti et al. 2020), and pollinators (Ziska et al. 2016; Crowley et al. 2021).

Studies considering the impact of eCO_2_ on plant–insect interactions have primarily focused on foliage-eating species (Robinson et al. 2012; Mayoral et al. 2023). The possible effects of eCO_2_ on pollinators, including bees, has been mentioned by very few authors (Ziska et al. 2016; Jamieson et al. 2017) and are far less understood. Given the great importance of plant–pollinator relationships, the effects of eCO_2_ on the nutritional quality of pollen, particularly in terms of elemental composition (stoichiometry), remains an important knowledge gap (Ziska et al. 2016; Crowley et al. 2021). In support of the nutrient dilution hypothesis, Ziska et al. (2016) identified increase in C:N ratios in the floral (anthers and pollen) tissue of *Solidago canadensis* (Canadian goldenrod) plants from North American museum collections spanning the period 1842–2014, coinciding with an increase in CO_2_ from ca. 280 to 398 ppm. Moreover, the authors confirmed this phenomenon in a field experiment, observing an increase in C:N ratios in the floral tissue of *S. canadensis* plants grown along a continuous CO_2_ gradient (from 280 to 500 ppm) over two years. The authors discussed these changes in the context of possible negative effects on bee health and fitness. However, *S. canadensis* serves as a food source for palynivores only in late summer and autumn in the northern hemisphere (Inoue and Takasaki 2016). Furthermore, although *S. canadensis* may be a proxy for herbaceous plants, its accuracy in representing tree or shrub species regarding element allocation to different tissues and products remains uncertain. Trees are important to consider, as they can display great plasticity in adjusting their stoichiometry to capitalise on eCO_2_ enrichment in capturing and allocating C for the growth of different tissues (Dror and Klein 2022). This flexibility can affect the nutritional value of plant material for consumers. Consideration of trees is also crucial because tree pollen, including that from wind-pollinated trees, is an essential food resource for many species of bees (Splitt et al. 2021; Cunningham-Minnick et al. 2023). For instance, oak pollen serves as a preferred source of nutritionally balanced food for both solitary bees, e.g. *Osmia bicornis* (red mason bee) (Splitt et al. 2021), and eusocial bees, e.g. *Apis mellifera* (honey bee) (Lau et al. 2018). These species provide important ecological and agricultural services by pollinating both natural flora and cultivated crops. Hence, the plant species that these bees gather pollen from are also valuable model organisms for studying the broader effects of climate change on pollinators.

Against this background, we investigated the effects of eCO_2_ on the variability in the concentrations and proportions of 12 chemical elements (macro- and micronutrients)—carbon (C), nitrogen (N), phosphorous (P), sulphur (S), potassium (K), sodium (Na), calcium (Ca), magnesium (Mg), copper (Cu), zinc (Zn), iron (Fe), and manganese (Mn)—in pollen attached to pollen-bearing flowers of *Q. robur* collected from the BIFoR-FACE facility. We considered basic elements that make up the physical components of organisms (C and N), bulk biological elements (e.g., P, S, K, Na, Ca, Mg), and trace elements, including Cu, Zn, Fe and Mn that, for instance, act as cofactors of enzymes (Sterner and Elser 2002; Kaspari 2021). We assessed the potential consequences of changes in pollen stoichiometry due to eCO_2_ that may subsequently lead to shifts in pollen nutritional quality for bees. To this end, we calculated the trophic stoichiometric mismatch by means of the trophic stoichiometric ratio (TSR) (Filipiak and Filipiak 2022). The TSR is a mathematical representation of the discrepancy between consumer nutrient demand and environmental nutrient supply and is a useful tool for identifying elements that (co-)limit consumers due to their scarcity in food (Filipiak and Filipiak 2022). We hypothesised that (1) compared to oak pollen produced under ambient CO_2_ levels (aCO_2_), oak pollen exposed to eCO_2_ would exhibit changes in multi-element stoichiometry, (2) the highest shift in elemental ratios would be observed for the C:N ratio, and (3) eCO_2_-induced changes in pollen stoichiometry would result in reduced pollen nutritional quality for bees, i.e., in stoichiometric mismatch between pollen and bees. We discuss the results in the context of the impact of eCO_2_ on bee health and function through the application of a stoichiometric mismatch.

## Materials and methods

### Facility

**Fig. 1:**
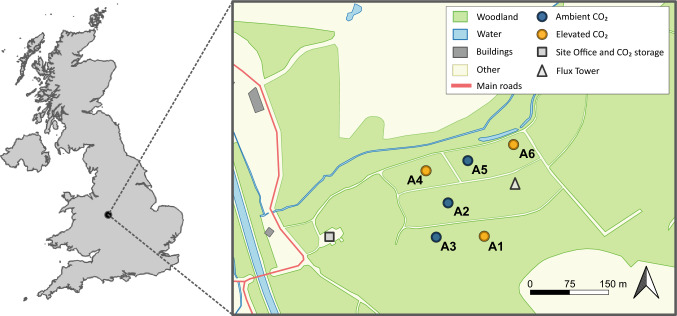
Study area location within the Birmingham Institute of Forest Research (BIFoR) Free Air Carbon Enrichment (FACE) facility, Staffordshire, United Kingdom. Arrays (A) 1, 4, and 6 are eCO_2_ treatments receiving + 150 ppm CO_2_ above ambient. Arrays 2, 3, and 5 are controls (aCO_2_) with ambient air (412 ppm during this study). The array points do not represent the actual dimensions of the CO_2_-enriched arrays. The map was created using QGIS version 3.32.2-Lima (Quantum GIS Development Team 2023). The land cover characteristics were derived from the Open Data website Ordnance Survey OS VectorMap® District (Ordnance Survey 2023)

The BIFoR-FACE facility is located in Staffordshire, United Kingdom (52°47ʹ58ʺ N, 2°18ʹ15ʺ W) (Fig. 1). The site is situated in a temperate deciduous forest dominated by pedunculate oak (*Q. robur*). The experimental area of the facility consists of six arrays approximately 30 m in diameter with a 25-m tall canopy. Three eCO_2_/treatment arrays were enriched with CO_2_ (+ 150 ppm above the ambient CO_2_ concentration). Three aCO_2_/control arrays, with identical infrastructure to that of the eCO_2_ arrays, were maintained under ambient atmospheric CO_2_ (approximately 412 ppm at the time of the measurements). The ~ 560 ppm (i.e., 412 ppm + 150 ppm) concentration used is designed to simulate atmospheric CO_2_ levels projected to occur by 2050. This scenarios represents a doubling from pre-industrial levels and provide valuable insights into near-term forest responses to elevated CO_2_. The FACE experiment started in April 2017 and will continue until at least 2031. Enrichment with CO_2_ occurred only during each growing season, from early April to late October. The fumigation occurs during daytime, and is discontinued at night. Moreover, the fumigation is stopped during high wind speed (15 min average wind speed, > 8 m/s). Full site details can be found in Hart et al. (2020) and MacKenzie et al. (2021).

### Plant material collection

In each experimental array, one oak tree was accessed (between 10 and 30 m above ground level) using a canopy access system. At the end of April 2022, approximately 10–15 inflorescences with pollen-bearing anthers per tree were collected. Importantly, the term “per tree” corresponds to “per array” as in each array only one tree was inspected. Therefore, the material collected from each tree represents a sample, i.e., a true replicate. This material consisted of inflorescences (subsamples), from which analytical subsamples were created. For further details on the material collection, please see Fig. S1. The collection dates coincided with the time of pollen formation and pollination. Between 0.4 and 2.4 g of dry inflorescence mass per tree was collected for analysis. After collection, the subsamples were dried at 60 °C for 24 h and then stored at  – 20 °C until shipment. All the subsamples were shipped to the Institute of Environmental Sciences, Jagiellonian University, Kraków, Poland, where they were stored at  – 40 °C until analysis. Immediately prior to analysis, the subsamples were dried in a vacuum dryer (80 °C, < 20 mbar, 24 h) to obtain the dry mass. The extraction of pollen poses a challenge and was not feasible in this instance. Although oak trees are wind pollinated, a method involving the use of pollination bags, which is commonly employed for pollen collection, was not applicable in our case due to restricted access to the canopy. Nevertheless, we believe that the material collected is a reliable representation of the pollen potentially collected by palynivores (a similar approach was used previously by (Welti and Kaspari 2021)).

### Chemical analysis

The inflorescences were prepared by detaching pollen-bearing flowers from the peduncle. For elemental analysis, we created analytical subsamples. For C, N, and S, a relatively small sample (i.e., 10 mg dry mass) is required for analysis, whereas for all other elements, the analytical subsamples needed to have a minimum of 150 mg dry mass. For this reason, for C, N, and S analyses, it was possible to create analytical subsamples from separate inflorescences, allowing for the collection of 6–10 analytical subsamples from 6 to 10 inflorescences per tree. Subsequently, the residual material, approximately 150 mg dry mass per sample, comprising pooled pollen-bearing flowers (3–5 samples per tree), was used for P, K, Na, Ca, Mg, Cu, Zn, Fe, and Mn analyses. All the analytical subsamples were ground manually using a porcelain mortar and were then freeze-dried. The C, N and S concentrations were measured in 49 analytical subsamples using a Vario EL III automatic CHNS analyser. For all the other elements, the material was digested in a 4:1 solution of nitric acid (70%) and perchloric acid (65%) using a hotplate. After digestion, the analytical subsamples were supplemented with distilled and deionised (Type 1) water (18 mΩ), and the P concentrations were determined by colorimetry (FIA: MLE FIA flow injection analyser). K, Na, Ca, Mg, Cu, Zn, Fe, and Mn concentrations were determined via atomic absorption spectrometry (Perkin Elmer AAnalyst 200 and Perkin Elmer AAnalyst 800) in 24 analytical subsamples. For C, N, P, and S, the results are expressed as a percentage of dry mass, whereas all the other elements are reported as milligrams per kilogram of dry mass. To determine the degree of analytical precision, four blanks were used for each analysis, along with sulphanilic acid as the reference material for the C, N, and S analyses and four different reference materials for P, K, Na, Ca, Mg, Cu, Zn, Fe, and Mn (National Institute of Standards and Technology USA: NIST SRM 1575a – trace elements in pine needles; NIST SRM 1577c – bovine liver; National Research Council of Canada: DOLT-5: Dogfish Liver Certified Reference Material for Trace Metals and other Constituents; and BOVM-1: Bovine Muscle Certified Reference Material for Trace Metals and other Constituents), which were examined with the analytical subsamples.

### Trophic stoichiometric ratio index calculation

To determine whether there was a potential stoichiometric mismatch between bees feeding on oak and pollen-bearing flowers (representing pollen provision), we calculated the trophic stoichiometric ratio (TSR; Filipiak and Filipiak 2022):

1 $${\text{TSR}}_{x} = \left( {C:X} \right)_{{{\text{food}}}} /\left( {C:X} \right)_{{{\text{consumer}}}}$$

where *C* is the carbon concentration and *X* is the concentration of element *x*—here, N, S, P, K, Na, Ca, Mg, Cu, Zn, Fe, and Mn. The index provides an indication of the limiting effect imposed by food (i.e., oak pollen-bearing flowers) on the consumer (i.e., bee) by nutritional imbalance. The assimilation efficiency of all the elements, except for carbon, is assumed to be at a maximum, i.e., 100%, whereas for carbon, it is assumed to be 25% due to loss through respiration. A TSR ≥ 1/0.25 (TSR ≥ 4) conservatively indicates a possible mismatch, and the higher the value is, the more severe the mismatch expected for the studied element. For more details on the TSR, please refer to Filipiak and Filipiak (2022).

When assessing the mismatch, we used the bee demand for elements gathered during larval development to construct a fully functional body comprising either the (i) adult *O. bicornis* body and cocoon (Filipiak 2019) or (ii) the adult *A. mellifera* body (Filipiak et al. 2017). The cocoon produced by *O. bicornis* is a secretion that forms an external, rigid, and strong protective cover for the larva during pupation and after pupation throughout subsequent seasons (i.e., summer, autumn, and winter) (Splitt et al. 2022). The cocoon encapsulates developing bee, serving as a barrier against external factors, (e.g., parasites). The larva secretes the cocoon, relying on matter stored in its body. This matter is consumed during larval development, assimilated into the larval body from pollen, and then allocated partly to the adult body and partly to the cocoon. Therefore, the cocoon, along with the body of the adult bee, constitutes the total production of matter necessary for the bee’s survival. Unlike *O. bicornis*, *A. mellifera* does not produce the typical robust and thick cocoon that protects against pathogens and environmental factors. The *A. mellifera* cocoon is very thin and fragile and is tightly integrated into the larval cell structure, making it susceptible to damage, tearing and changes in elemental composition during handling. Moreover it contains, in addition to bee secretions, embedded faeces produced throughout larval development and secreted once during cocoon production (Jay 1964). This characteristics makes it almost not feasible to obtain unaltered measurements of the chemical elements that make up the *A. mellifera* cocoon. Importantly, the mass of the *A. mellifera* cocoon relative to the total mass of the larva ready to pupate is not as large as that of *O. bicornis*. Additionally, there is currently no data available on the elemental composition of *A. mellifera* cocoons. Therefore, only the adult body, excluding the cocoon, was used to calculate the TSR for *A. mellifera*. The TSRs were calculated for each treatment separately (aCO_2_ and eCO_2_). Based on the number of true replicates obtained, 3 C:*X* ratios were used in the TSR numerator (i.e., food) for the aCO_2_ treatment, and 3 C:*X* ratios were used in the TSR numerator (i.e., food) for the eCO_2_ treatment. The C:*X* were the average values for each array. For the denominator (i.e., consumer), we used published data that included 30 C:*X* ratios for *O. bicornis* (Filipiak 2019) and 45 C:*X* ratios for *A. mellifera* (Filipiak et al. 2017). Consequently, for *O. bicornis,* the total calculated TSR values were 90 (3 × 30) and 90 (3 × 30) for the aCO_2_ and eCO_2_ treatments, respectively. For *A. mellifera,* the total number of calculated TSRs was 135 (3 × 45) for aCO_2_ and 135 (3 × 45) for eCO_2_.

### Statistical analysis

Our dataset had a total of *n* = 22 and *n* = 27 measurements for C, N, and S obtained for the aCO_2_ and eCO_2_ treatments, respectively. For the other elements (P, K, Na, Ca, Mg, Cu, Zn, Fe, Mn), we obtained *n* = 12 and *n* = 11 measurements, for the aCO_2_ and eCO_2_ treatments, respectively. The measurements were conducted on the analytical subsamples. All the statistical analyses were performed in R version 4.3.1 for Windows (R Core Team 2023). To determine whether there were significant differences in the elemental composition among the pollen-bearing flowers composing analytical subsamples from the different treatments (aCO_2_ vs eCO_2_), a permutational analysis of variance (PERMANOVA) was performed based on the Bray‒Curtis dissimilarity matrix with 999 permutations using the ‘adonis2’ function. In next step, to visualize the changes in multi-element stoichiometry of pollen-bearing flowers (analytical subsamples), we employed non-metric multidimensional scaling (NMDS) using the ‘metaMDS’ function. The analysis was conducted on elemental data with Bray‒Curtis dissimilarity as the distance measure, with set of number dimensions *k* = 2. To assess the relationship between CO_2_ treatments and the NMDS ordination, we used the ‘envfit’ Both PERMANOVA and NMDS analyses were performed using the ‘vegan’ package (Oksanen et al. 2022). For both PERMANOVA and NMDS, first, the elemental concentrations were normalised as elemental data were measured in different units. PERMANOVA and NMDS were not performed for the true replicates due to the small sample size (*n* = 3 for each treatment); instead, they were conducted for analytical subsamples. Given the nature of our data, we chose PERMANOVA and NMDS for their robustness in analysing multivariate differences. PERMANOVA served as the initial step to assess overall multivariate differences between treatments, while NMDS was used to visualize the variation in elemental composition among treatments.

To compare the concentrations of each element (C, N, S, P, K, Na, Ca, Mg, Cu, Zn, Fe, and Mn) and C:N, C:P, and N:P ratios between aCO_2_ and eCO_2_, we used general linear mixed models (LMMs) fitted with the R package ‘nlme’ (Pinheiro et al. 2023). To determine the appropriate normalization transformations for concentrations of each element, we used the ‘bestNormalize’ package (Peterson 2021), which identifies optimal transformations to improve the adherence of data to statistical assumptions. Carbon and nitrogen concentrations, as well as C:N ratios, were transformed using the Box–Cox transformation, while manganese concentrations were logarithmically transformed. The concentrations of each element, or the proportions of elements, were fitted to LMMs as the dependent variable against CO_2_ treatment as a fixed effect. To avoid pseudoreplication, the random effect was specified using (1 | array/subsample) to account for the nesting of subsamples (i.e., analytical subsamples) within arrays, as the subsamples collected from each array originated from an individual tree. For model validation, standardised residuals were examined for normality and homogeneity of variance using either a Q‒Q plot of residuals combined with the Shapiro‒Wilk test or Levene’s test. To assess the overall model fit, we calculated marginal (fixed effects) and conditional (random and fixed effects) coefficients of determination (R^2^) using the ‘r.squaredGLMM’ function from the ‘MuMIn’ package (Bartoń 2023). We employed a nonparametric Mann‒Whitney test to compare the TSR values calculated for each element between the aCO_2_ treatment and the eCO_2_ treatment.

The uniqueness of the BIFoR-FACE facility, among other forest FACE experiments operating worldwide (Norby et al. 2016), particularly for mature northern temperate forests (Hart et al. 2020), imposes constraints on material collection and research procedures to minimise additional impacts on the ecosystem. Due to the site enclosure and access system (Hart et al. 2020), our sampling was restricted to one individual *Quercus* tree per array, and for each tree, subsamples were collected and used in the analyses. Thus, we acknowledge that our study entails a level of pseudoreplication, given that the subsamples gathered from each array are derived from individual trees (Oksanen 2001). Nevertheless, each treatment consisted of *n* = 3 true replicates (i.e., trees); therefore, we tested the hypothesis regarding differences in elemental composition between the treatments (aCO_2_ vs. eCO_2_). Notably, large-scale CO_2_ enrichment experiments often involve a relatively low number of true replicates (see, e.g., Gardner et al. 2023). Thus, this limitation is not exclusive to our research; it is also applicable to other studies involving the collection of plant material in FACE facilities (Martins et al. 2021).

## Results

### Elemental composition

**Fig. 2:**
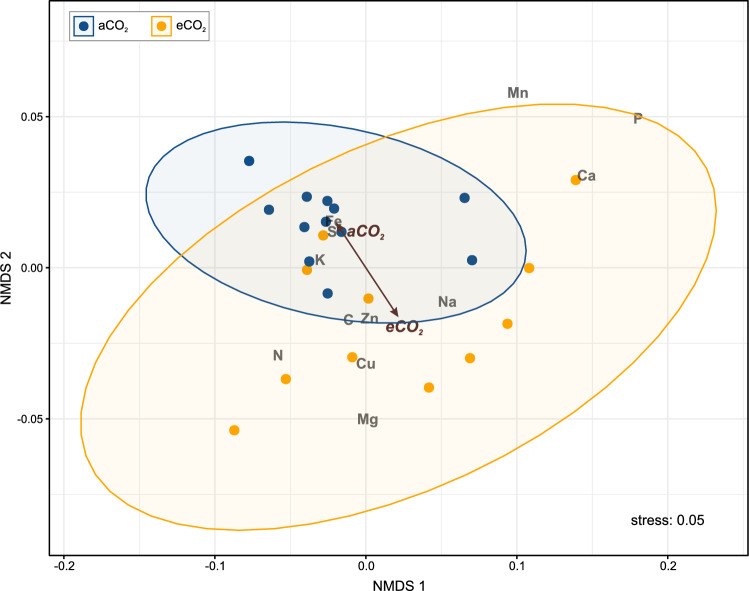
Non-metric multidimensional scaling (NMDS) ordination plot of multi-elemental stoichiometry of pollen-bearing flowers from pedunculate oak (*Quercus robur*) collected under ambient CO_2_ (aCO_2_) and elevated CO_2_ (eCO_2_) treatments. Points represent arrays and are color-coded by CO_2_ treatment (blue: aCO_2_, orange: eCO_2_). The brown arrows represent CO_2_ treatments, indicating the direction and magnitude of their influence on elemental composition. Elemental scores are denoted by text labels, and ellipses represent 95% confidence intervals around the centroid of each data cluster. A stress value of 0.05 indicates a goodness of fit

The concentrations of the elements measured in the oak pollen-bearing flowers were as follows: carbon (49.6–49.8%); nitrogen (4.7–4.8%); potassium (1.8–1.9%); phosphorus, magnesium, and calcium (0.3–0.4%); sulphur (ca. 0.2%); and manganese, sodium, iron, zinc, and copper (≤ 0.1%) (details in Table S1). The multi-element stoichiometries of the oak pollen-bearing flowers differed significantly between the treatments, although the relatively low variability in the data was explained by the model (PERMANOVA: R^2^ = 0. 158, pseudo-F_1,21_ = 3.96, *p* value = 0.04). Visualization of the multi-element stoichiometries of the oak pollen-bearing flowers using the two dimensions of the NMDS plot (stress = 0.05) revealed that each treatment exhibited partially distinct chemical characteristics. The S, K, and Fe elemental scores were associated with samples from aCO_2_ treatment, suggesting higher concentrations of these elements in aCO_2_ treatment. The Mg and Ca elemental scores were more associated with eCO_2_ treatment, suggesting higher concentrations of these elements under eCO_2_ conditions. The subsamples from the aCO_2_ treatment were more clustered together, while the subsamples from the eCO_2_ treatment were more variable in terms of elemental composition (Fig. 2).

Comparisons of single element concentrations using LMMs revealed significant differences in S (*p* value = 0.04) and close to significant differences in K (*p* value = 0.06) and Fe (*p* value = 0.06) (Table 1), exhibiting approximately 10%, 8%, and 16% higher concentrations, respectively, for the aCO_2_ treatment (Table S1). The marginal R^2^ values were relatively low for S, K, and Fe and very low for all the other elements (Table 1). On the other hand, the conditional R^2^ was relatively high for all the elements (R^2^ > 0.9), thus indicating that while fixed effects accounted for a small portion of the variance, random effects explained a substantial proportion of the variance in each model.

**Table 1:** Summary of the results of linear mixed model (LMM) analyses of the effect of CO_2_ treatment on the various elemental concentrations and ratios of pollen-bearing flowers obtained from pedunculate oak (*Quercus robur*) collected from ambient and elevated CO_2_ treatment arrays at the BIFoR-FACE facility

Response variable		Estimate	SE	df	*t* value	*p* value	R^2^m	R^2^c
C	Intercept	3.76	0.41	43	9.27	< 0.0001		
	CO_2_	0.40	0.57	4	0.70	0.5	0.03	0.91
N	Intercept	3.84	0.59	43	6.48	< 0.0001		
	CO_2_	0.36	0.84	4	0.43	0.7	0.03	0.97
P	Intercept	0.35	0.09	17	3.69	0.002		
	CO_2_	– 0.02	0.13	4	– 0.17	0.9	0.004	0.95
S	Intercept	0.21	0.01	43	40.34	< 0.0001		
	CO_2_	– 0.02	0.01	4	– 2.89	0.04	0.21	0.91
K	Intercept	19,254.03	432.36	17	44.53	< 0.0001		
	CO_2_	– 1568.71	619.32	4	– 2.53	0.06	0.31	0.93
Na	Intercept	328.53	42.73	17	7.69	< 0.0001		
	CO_2_	– 52.08	60.84	4	– 0.86	0.4	0.07	0.92
Ca	Intercept	3621.62	605.54	17	5.98	< 0.0001		
	CO_2_	36.16	859.42	4	0.04	1.0	0.0002	0.93
Mg	Intercept	3856.04	127.34	17	30.28	< 0.0001		
	CO_2_	228.92	181.75	4	1.26	0.3	0.12	0.92
Cu	Intercept	22.67	1.25	17	18.18	< 0.0001		
	CO_2_	– 1.59	1.78	4	– 0.89	0.4	0.06	0.91
Zn	Intercept	67.08	2.84	17	23.65	< 0.0001		
	CO_2_	– 3.08	4.04	4	– 0.76	0.5	0.05	0.91
Fe	Intercept	118.19	5.75	17	20.54	< 0.0001		
	CO_2_	– 20.66	8.20	4	– 2.52	0.06	0.38	0.95
Mn	Intercept	3.74	0.08	17	47.71	< 0.0001		
	CO_2_	– 0.11	0.11	4	– 0.95	0.4	0.10	0.94
C:N	Intercept	3.95	0.58	17	6.76	< 0.0001		
	CO_2_	– 0.16	0.83	4	– 0.19	0.9	0.01	0.95
C:P	Intercept	151.13	43.61	17	3.47	0.003		
	CO_2_	55.16	61.75	4	0.89	0.42	0.11	0.97
N:P	Intercept	14.31	5.02	17	2.85	0.01		
	CO_2_	6.60	7.10	4	0.93	0.41	0.12	0.97

Each element was studied separately, and the model fit was evaluated using marginal (R^2^m) and conditional (R^2^c) R-squared values. The random effect accounted for the nesting of subsamples within arrays, as the subsamples collected from each array originated from an individual tree

### Trophic stoichiometric ratio

**Fig. 3:**
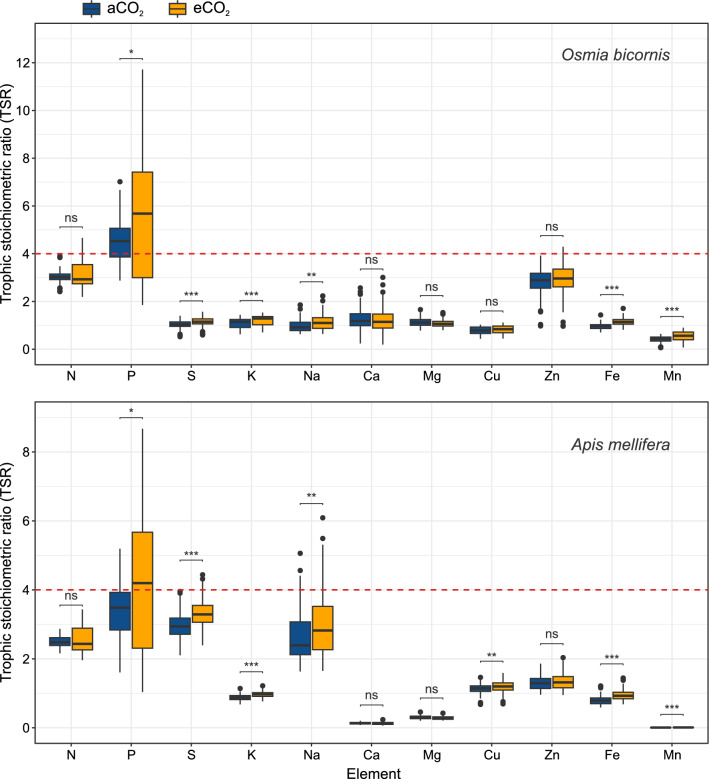
The trophic stoichiometric ratio (TSR) serves as a proxy for understanding the limiting effects of oak pollen-bearing flowers on the growth and development of *Osmia bicornis* (upper panel) and *Apis mellifera* (lower panel). The boxplots show the median and the first and third quartiles; whiskers extend to values ≤ 1.5 times the interquartile range of the box; circles show outliers. The red line indicates the threshold value of TSR = 4, and a TSR ≥ 4 indicates a limitation on bee production due to the scarcity of the element in the studied samples. The stoichiometric mismatches experienced by bees feeding on pollen from ambient CO_2_ (aCO_2_; blue) treatment were compared with those imposed by pollen from elevated CO_2_ (eCO_2_; orange) treatment (Mann‒Whitney test, significance levels: ns – not significant at *p* > 0.5, *p < 0.5, **p < 0.1, ***p < 0.01)

The stoichiometric mismatches, represented by the TSR values for *O. bicornis* bees, were significantly higher under eCO_2_ than aCO_2_ treatments for the P, S, K, Na, Fe, and Mn elements (Fig. 3 and Table 2). Only for phosphorus did the TSR exceed 4, indicating a limiting effect of stoichiometric mismatch on *O. bicornis*. These values were significantly greater for eCO_2_. The TSRs calculated for *A. mellifera* were significantly higher under eCO_2_ than aCO_2_ treatments for P, S, K, Na, Cu, Fe, and Mn elements (Fig. 3). Similar to *O. bicornis*, the TSR value for *A. mellifera* exceeded 4 only for phosphorus, and this occurred exclusively under the eCO_2_ treatment (Table 2).

**Table 2:** Trophic stoichiometric ratio (TSR) values (median, 25th percentile, 75th percentile) of eleven non-carbon elements

Element	Treatment	*Osmia biconis*			*Apis mellifera*		
		Median	25th percentile	75th percentile	Median	25th percentile	75th percentile
N	aCO_2_	3.02	2.92	3.15	2.48	2.39	2.61
	eCO_2_	2.93	2.74	3.54	2.43	2.26	2.89
P	aCO_2_	4.53	3.86	5.07	3.48	2.84	3.93
	eCO_2_	5.68	2.99	7.42	4.20	2.31	5.67
S	aCO_2_	1.03	0.96	1.14	2.94	2.71	3.18
	eCO_2_	1.15	1.06	1.27	3.29	3.06	3.55
K	aCO_2_	1.15	0.91	1.25	0.87	0.82	0.93
	eCO_2_	1.29	1.02	1.37	0.97	0.91	1.02
Na	aCO_2_	0.91	0.78	1.13	2.39	2.12	3.07
	eCO_2_	1.10	0.88	1.32	2.82	2.26	3.52
Ca	aCO_2_	1.18	0.98	1.49	0.13	0.11	0.15
	eCO_2_	1.15	0.89	1.47	0.12	0.10	0.15
Mg	aCO_2_	1.12	1.00	1.24	0.30	0.26	0.34
	eCO_2_	1.06	0.97	1.17	0.29	0.25	0.32
Cu	aCO_2_	0.79	0.66	0.93	1.14	1.05	1.22
	eCO_2_	0.84	0.69	0.97	1.20	1.09	1.30
Zn	aCO_2_	2.89	2.56	3.18	1.29	1.14	1.43
	eCO_2_	2.96	2.61	3.36	1.32	1.16	1.48
Fe	aCO_2_	0.97	0.87	1.03	0.79	0.70	0.86
	eCO_2_	1.14	1.05	1.24	0.93	0.84	1.03
Mn	aCO_2_	0.44	0.34	0.51	0.006	0.005	0.007
	eCO_2_	0.56	0.40	0.72	0.009	0.006	0.010

TSR values were calculated as a proxy of the stoichiometric mismatches between bee consumer, either *Osmia bicornis* or *Apis mellifera*, and the pollen-bearing flowers obtained from pedunculate oak (*Quercus robur*) representing bee food. Plant material was collected from ambient (aCO_2_) and elevated (eCO_2_) treatments at the Birmingham Institute of Forest Research (BIFoR) Free Air Carbon Enrichment (FACE) facility

## Discussion

Utilising the BIFoR-FACE facility, which operates under natural and fully open-air conditions, we demonstrated that five-year long atmospheric CO_2_ enrichment changed the stoichiometry of pollen-bearing oak flowers. It is plausible that these changes could negatively affect palynivores. Changes in stoichiometry were mainly driven by lower concentrations of S, K and Fe in flowers collected at aCO_2_ and higher concentrations of Mg in flowers collected at eCO_2_, with a lesser effect on the concentrations of the other elements studied. The overall strength of the stoichiometric mismatch between the two bee species (*O. bicornis* and *A. mellifera*) and pollen was greater for trees experiencing eCO_2_ than for those experiencing aCO_2_ concentrations. However, only certain elements exhibited stoichiometric mismatch values high enough to be limiting for bees. Specifically, the scarcity of P in eCO_2_ pollen could limit *O. bicornis*, while the scarcity of P and potentially S in eCO_2_ pollen could limit *A. mellifera*. Increasing atmospheric CO_2_ concentrations affect ecosystems (Tylianakis et al. 2008; Calvin et al. 2023), leading to alterations in the nutritional composition of plant tissues and products (Loladze 2014; Ziska et al. 2016). In this context, it is important to explore the impact of eCO_2_ on the quality of pollen, which serves as a food source not only for bees, but also for other pollinators (e.g., pollen wasps or hoverflies) (Ollerton 2021; Lau et al. 2022).

### Oak pollen-bearing flower stoichiometry under elevated CO_2_

In support of our first hypothesis, we observed that compared to oak trees exposed to aCO_2_ levels, oak trees exposed to eCO_2_ exhibited changes in the elemental stoichiometry of pollen-bearing flowers. Specifically, we observed lower levels of K, S, and Fe in the pollen from the enriched CO_2_ arrays. K is an indispensable nutrient vital for plant functions, such as regulating water balance, supporting photosynthesis, activating enzymes, bolstering stress resilience, and various other physiological processes (Sardans and Peñuelas 2015). K is also crucial for pollen germination (Hasanuzzaman et al. 2018). Regarding S and Fe, S plays a vital role in protein synthesis and structural integrity, and Fe is essential for energy production and enzyme activation (Hänsch and Mendel 2009). Together, Fe and S form essential components of Fe–S clusters, which are present across all domains of life and are important for processes such as respiration, photosynthesis, and metabolism (Lill and Mühlenhoff 2006). For example, Fe is essential for the functioning of mitochondria in pollen and ultimately for yield (Morrissey and Guerinot 2009). Consequently, the observed decreases in K, S and Fe contents in oak pollen may affect pollen fertility and plant reproductive success.

To date, only a few studies have determined the effects of eCO_2_ on pollen quality (i.e., protein or metabolite content) and quantity (i.e., production), with contrasting results (Crowley et al. 2021 and references therein). To our knowledge, this is the first study in which the elemental composition of pollen-bearing flowers of a tree was studied; thus, it is not possible to discuss the results in the context of similar studies. Nevertheless, the effects of eCO_2_ on plant physiology and the concentrations of various elements have already been demonstrated for different plant tissues (Dong et al. 2018; Peñuelas et al. 2020). For instance, changes in elemental stoichiometry were observed for red oak (*Quercus rubra*) leaves and Norway spruce (*Picea abies*) needles collected from young trees (4 and 6 years old, respectively) after one year of fumigation in open-top chambers (ambient + 350 ppm of CO_2_). Specifically, significantly lower concentrations of K, Ca, Mn, and N were measured in both oak leaves and spruce needles collected from the elevated treatment than in those collected from the ambient treatment (Le Thiec et al. 1995). Moreover, lower concentrations of Mg were recorded in the spruce needles, but not oak leaves from the elevated treatment (Le Thiec et al. 1995). In our study, Mg concentrations were 6% higher under eCO_2_ than aCO_2_ treatments, although this difference was not statistically significant. Interestingly, Welti et al. (2020) did not observe changes in Mg concentrations in plants collected over more than three decades in the Kansas prairie, while declines were observed for N, P, K, and Na. These results were associated with increased atmospheric CO_2_ levels. The lack of change, or even an increase, in Mg concentrations under eCO_2_ may be due to physiological adaptations, considering that Mg is a key component of chlorophyll, which is essential for photosynthesis. Notably, the shifts in elemental stoichiometry resulting from eCO_2_ levels may manifest to varying extents and may differ across various plant species and their respective tissues and products (Dong et al. 2018; Guo et al. 2021). Moreover, changes may depend on latitude (Peñuelas et al. 2020) or other factors related to global changes (e.g., drought, temperature, fertilisation) (Carvalho et al. 2020; Ainsworth and Long 2021). However, what remains certain is that an increase in atmospheric CO_2_ levels will inevitably induce changes in the elemental stoichiometry of plants. These alterations not only affect physiological processes, but also subsequently impact other trophic levels and the functioning of ecosystems (Kaspari 2021).

In the present study, we did not observe changes in the C:N ratio; thus, the second hypothesis was not supported. The absence of the anticipated change in C:N ratios could stem from various factors, including tree physiology, soil N transformations, and atmospheric N deposition. Similarly, as in our study, no overall effects of CO_2_ were detected for green upper canopy leaves from a mature *Eucalyptus* forest over 5 years of treatment in a FACE experiment (ambient + 150 ppm CO_2_), with no differences in C:N or N:P (Crous et al. 2019). As for previous studies from BIFoR-FACE, no differences in foliar N concentrations were observed between the aCO_2_ and eCO_2_ treatments after 3 and 4 years of fumigation (Gardner et al. 2022a, b). However, a greater average leaf mass per area, denoted as leaf thickness (Gardner et al. 2022b), and an approximately 10% greater oak litterfall (communicated personally by S. Ullah) were noted under eCO_2_. In parallel, soils from the aCO_2_ and eCO_2_ treatments at BIFoR-FACE had similar concentrations of K, Mg, Ca, and S, but higher concentrations of C, N, Na, and Cl under eCO_2_ (Sgouridis et al. 2023). Interestingly, the forests at BIFoR-FACE receive moderately high atmospheric N deposition (∼22 kg N/ha/yr), which is considered sufficient to prevent nitrogen limitation within the current ecosystem (Rennenberg and Dannenmann 2015; Gardner et al. 2022a). Taken together, despite differing soil chemical conditions, both leaves (Gardner et al. 2022a, b) and pollen (present study) may retain nitrogen similarly under both aCO_2_ and eCO_2_ treatments due to sufficient atmospheric N deposition (Rennenberg and Dannenmann 2015), soil N availability and N transformation (Sgouridis et al. 2023). However, there was an overall increase in leaf mass production and lower K, S, and Fe concentrations in pollen under eCO_2_, suggesting that other resources (i.e., elements other than N) may not be allocated uniformly across the different CO_2_ treatments. Consequently, it is probable that, under eCO_2,_ the maintenance of certain properties and functions within specific tissues is linked to the consistent maintenance of certain key elements (e.g., N, a key element for protein formation) at a constant level. However, this maintenance may be compensated for by alterations in the levels of other elements (e.g., micronutrients). Yet, the phenomenon of sustained nitrogen levels might change under prolonged CO_2_ emissions, as suggested by the findings of previous studies (Peñuelas et al. 2020), or it could be specific to certain geographical areas (Gardner et al. 2022a; Sgouridis et al. 2023).

### Possible effects on bees

Our findings revealed treatment-related variations in the elemental composition of pollen-bearing flowers, primarily attributed to S, K, and Fe. While K is an essential nutrient for overall plant health and productivity, excessive K concentrations in the diet of plant consumers can be limiting and may result in health issues, potentially leading to mortality (Kaspari 2020). In our study, we observed lower levels of K in the eCO_2_ treatment than under aCO_2_, which might suggest a potential benefit for herbivores and palynivores. However, it appears that a balanced potassium-to-sodium (K:Na) ratio, rather than the K concentration itself, is necessary for the optimal functioning of organisms, including bees (Cairns et al. 2021; Filipiak et al. 2023). Regarding S and Fe, S is a key component of various proteins and amino acids (e.g., methionine and cysteine) (da Silva and Williams 2001; Lau et al. 2022), whereas Fe serves as a cofactor of several enzymes in insects, e.g., those involved in cellular respiration (Gorman 2023). Although the understanding of how these elements affect bee well-being and health is limited, some authors propose that S is linked to longevity and overall performance (Lau et al. 2022), whereas others propose that Fe is involved in defence mechanisms against pathogens in insects (Hrdina and Iatsenko 2022). Interestingly, the overall pattern of elemental partitioning between different components within the elemental budget of *O. bicornis* bees revealed that both Fe and S are primarily concentrated in the excreta and are assimilated to a low degree compared to other elements (Filipiak et al. 2021). Hence, the relative concentrations of these elements in pollen may not be as critical for bee health and fitness as the concentrations of other elements.

Our third hypothesis, related to stoichiometric mismatch, was partially supported, as our calculations suggested that the scarcity of P and S, but not other elements, in CO_2_-affected pollen may be limiting for bees. Specifically, by adopting a conservative approach in which TSR values exceeding a threshold of 4 indicate limiting effects of the studied element (with assimilation efficiency of 100%; see Filipiak and Filipiak (2022) for more details), our findings suggest that *O. bicornis* could face limitations in the availability of P, with these limitations being more pronounced under eCO_2_ conditions. However, the assimilation efficiency is expected to be lower than 100%. Only one study has presented preliminary estimations of the assimilation efficiencies of different elements from pollen consumed during larval development to bee bodies and cocoons (Filipiak et al. 2021). Filipiak et al. (2021) found that assimilation efficiencies differ for various elements, ranging from approximately 30–35% for S, 50–60% for Na, 55–70% for P, to 65–70% for Zn. These efficiencies might be potentially influenced by pollen type, pollen contamination, or the composition of symbionts in the gut. Given that Filipiak et al. (2021) is the only study on this topic and that no such data exist for *A. mellifera*, we conservatively and arbitrarily assumed an assimilation efficiency of 75% instead of 100%. Consequently, the limiting effects on *O. bicornis* were also observed for N but only for pollen under aCO_2_. Regarding *A. mellifera*, if the assimilation efficiency was 75%, limiting effects could be expected for P in both treatments, with more pronounced limitations in the eCO_2_ treatment. Additionally, S could become limiting, but only for pollen collected from the eCO_2_ treatment. Overall, changes in pollen stoichiometry would have the greatest effects on P limitation. Considering that P is a ubiquitous element essential for nucleic and ribonucleic acids, energetic nucleotides (i.e., ATP), and phospholipids, the limited availability of this element may have negative effects on bee growth, development, health, and fitness (Filipiak et al. 2022; Isanta‐Navarro et al. 2022).

Pollinators face numerous challenges due to climate change (Robinson et al. 2012; Dicks et al. 2021). The present study has identified that reduced diet quality can be added to the increasing list of negative impacts. This area of research is still in its infancy, thus it is important to continue investigating how eCO_2_ and the resulting changes in the stoichiometry of floral rewards (pollen and nectar) will affect the health and fitness of bees, their interactions with plants, the functioning of bee populations and communities, and ultimately the ecosystem services they provide. Moreover, alterations in pollen stoichiometry have been proven to have negative effects on bee development and growth (Filipiak et al. 2022). Further studies are crucial for elucidating the influence of changes in pollen nutrient quality resulting from anthropogenic greenhouse gas emissions on bee health and will broaden our understanding of pollinator dynamics in response to global climate change.

## Conclusions

Atmospheric CO_2_ enrichment over five years can influence the stoichiometry of pollen-bearing flowers collected from mature oaks. The observed changes in elemental composition, specifically, the lower concentrations of S, K, and Fe, along with possible colimiting effects caused by multielement stoichiometric mismatch under eCO_2_ treatment, may reduce the nutritional quality of pollen. Our first hypothesis that exposure to eCO_2_ would result in changes in the multi-element stoichiometry of pollen was supported by the evidence of altered multielement stoichiometry under eCO_2_, with different elements behaving in different ways. However, the second hypothesis, that the highest shift in elemental ratios would be observed for the C:N ratio, was not confirmed. Regarding the third hypothesis, that eCO_2_-induced changes in pollen stoichiometry result in reduced pollen nutritional quality for bees, we identified potential limitations of P scarcity for *O. bicornis* and P and S scarcity for *A. mellifera* consuming pollen produced under eCO_2_. In conclusion, our study provides insights into the relationships among environmental changes, plant stoichiometry, and potential effects on the well-being of bees. Moreover, we show that in an era of global climate change, understanding the stoichiometric constraints on pollinators is of paramount importance. Therefore, further investigations, including feeding experiments and field data, are essential to gain a more comprehensive understanding of how these changes may affect bee growth, health, and ultimately population in a changing environment.

## Supplementary Information

Below is the link to the electronic supplementary material.

Supplementary file1 (DOCX 581 KB)

## Data Availability

Data are available upon request from the authors.

## References

[CR1] Ainsworth EA, Long SP (2021) 30 years of free-air carbon dioxide enrichment (FACE): what have we learned about future crop productivity and its potential for adaptation? Glob Change Biol 27:27–49. 10.1111/gcb.15375 10.1111/gcb.15375 33135850

[CR2] Bartoń K (2023) _MuMIn: multi-model inference_. R package version 1.47.5. https://CRAN.R-project.org/package=MuMIn

[CR3] Cairns SM, Wratten SD, Filipiak M, Veronesi ER, Saville DJ, Shields MW (2021) Ratios rather than concentrations of nutritionally important elements may shape honey bee preferences for ‘dirty water.’ Ecol Entomol 46:1236–1240. 10.1111/een.13067

[CR4] Calvin K, Dasgupta D, Krinner G, Mukherji A, Thorne P, Trisos C, Romero J, Aldunce P, Barrett K, Blanco G, Cheung WWL, Connors SL, Denton F, Diongue-Niang A, Dodman D, Garschagen M, Geden O, Hayward B, Jones C, Jotzo F, Krug T, Lasco R, Lee JY, Masson-Delmotte V, Meinshausen M, Mintenbeck K, Mokssit A, Otto FEL, Pathak M, Pirani A, Poloczanska E, Pörtner HO, Revi A, Roberts DC, Roy J, Ruane AC, Skea J, Shukla PR, Slade R, Slangen A, Sokona Y, Sörensson AA, Tignor M, Vuuren Dv, Wei YM, Winkler H, Zhai P, Zommers Z (2023) IPCC, 2023: climate change 2023: synthesis report. Contribution of working groups I, II and III to the sixth assessment report of the intergovernmental panel on climate change. IPCC, Geneva, Switzerland

[CR5] Carvalho JM, Barreto RF, Prado RDM, Habermann E, Martinez CA, Branco RBF (2020) Elevated [CO_2_] and warming increase the macronutrient use efficiency and biomass of *Stylosanthes capitata* Vogel under field conditions. J Agron Crop Sci 206:597–606. 10.1111/jac.12398

[CR6] Crous KY, Wujeska-Klause A, Jiang M, Medlyn BE, Ellsworth DS (2019) Nitrogen and phosphorus retranslocation of leaves and stemwood in a mature eucalyptus forest exposed to 5 years of elevated CO_2_. Front Plant Sci 10:664. 10.3389/fpls.2019.00664 31214212 10.3389/fpls.2019.00664 PMC6554339

[CR7] Crowley LM, Sadler JP, Pritchard J, Hayward SAL (2021) Elevated CO_2_ impacts on plant–pollinator interactions: a systematic review and free air carbon enrichment field study. Insects 12:512. 10.3390/insects12060512 34206033 10.3390/insects12060512 PMC8227562

[CR8] Cunningham-Minnick M, Milam J, Kane B, Roberts H, King DV (2023) Abundant, distinct, and seasonally dynamic bee community in the canopy-aerosphere interface above a temperate forest. Ecol Evol 13:e9739. 10.1002/ece3.9739 36818539 10.1002/ece3.9739 PMC9929519

[CR9] da Silva JJRF, Williams RJP (2001) The biological chemistry of the elements. The inorganic chemistry of life. Oxford University Press, Oxford

[CR10] Dicks LV, Breeze TD, Ngo HT, Senapathi D, An J, Aizen MA, Basu P, Buchori D, Galetto L, Garibaldi LA, Gemmill-Herren B, Howlett BG, Imperatriz-Fonseca VL, Johnson SD, Kovacs-Hostyanszki A, Kwon YJ, Lattorff HMG, Lungharwo T, Seymour CL, Vanbergen AJ, Potts SG (2021) A global-scale expert assessment of drivers and risks associated with pollinator decline. Nat Ecol Evol 5:1453–1461. 10.1038/s41559-021-01534-9 34400826 10.1038/s41559-021-01534-9

[CR11] Dong J, Gruda N, Lam SK, Li X, Duan Z (2018) Effects of elevated CO_2_ on nutritional quality of vegetables: a review. Front Plant Sci 9:924. 10.3389/fpls.2018.00924 30158939 10.3389/fpls.2018.00924 PMC6104417

[CR12] Dror D, Klein T (2022) The effect of elevated CO_2_ on aboveground and belowground carbon allocation and eco-physiology of four species of angiosperm and gymnosperm forest trees. Tree Physiol 42:831–847. 10.1093/treephys/tpab136 34648020 10.1093/treephys/tpab136

[CR13] Filipiak M (2019) Key pollen host plants provide balanced diets for wild bee larvae: a lesson for planting flower strips and hedgerows. J Appl Ecol 56:1410–1418. 10.1111/1365-2664.13383

[CR14] Filipiak M, Filipiak ZM (2022) Application of ionomics and ecological stoichiometry in conservation biology: nutrient demand and supply in a changing environment. Biol Conserv 272:109622. 10.1016/j.biocon.2022.109622

[CR15] Filipiak M, Kuszewska K, Asselman M, Denisow B, Stawiarz E, Woyciechowski M, Weiner J (2017) Ecological stoichiometry of the honeybee: pollen diversity and adequate species composition are needed to mitigate limitations imposed on the growth and development of bees by pollen quality. PLoS One 12:e0183236. 10.1371/journal.pone.0183236 28829793 10.1371/journal.pone.0183236 PMC5568746

[CR16] Filipiak M, Woyciechowski M, Czarnoleski M (2021) Stoichiometric niche, nutrient partitioning and resource allocation in a solitary bee are sex-specific and phosphorous is allocated mainly to the cocoon. Sci Rep 11:652. 10.1038/s41598-020-79647-7 33436811 10.1038/s41598-020-79647-7 PMC7804283

[CR17] Filipiak ZM, Denisow B, Stawiarz E, Filipiak M (2022) Unravelling the dependence of a wild bee on floral diversity and composition using a feeding experiment. Sci Total Environ 820:153326. 10.1016/j.scitotenv.2022.153326 35074369 10.1016/j.scitotenv.2022.153326

[CR18] Filipiak ZM, Ollerton J, Filipiak M (2023) Uncovering the significance of the ratio of food K:Na in bee ecology and evolution. Ecology 104:e4110. 10.1002/ecy.4110 37232411 10.1002/ecy.4110

[CR19] Gardner A, Ellsworth DS, Crous KY, Pritchard J, MacKenzie AR (2022a) Is photosynthetic enhancement sustained through three years of elevated CO_2_ exposure in 175-year-old *Quercus robur*? Tree Physiol 42:130–144. 10.1093/treephys/tpab090 34302175 10.1093/treephys/tpab090 PMC8754963

[CR20] Gardner A, Ellsworth DS, Pritchard J, MacKenzie AR (2022b) Are chlorophyll concentrations and nitrogen across the vertical canopy profile affected by elevated CO_2_ in mature *Quercus* trees? Trees 36:1797–1809. 10.1007/s00468-022-02328-7

[CR21] Gardner A, Jiang M, Ellsworth DS, MacKenzie AR, Pritchard J, Bader MKF, Barton CVM, Bernacchi C, Calfapietra C, Crous KY, Dusenge ME, Gimeno TE, Hall M, Lamba S, Leuzinger S, Uddling J, Warren J, Wallin G, Medlyn BE (2023) Optimal stomatal theory predicts CO_2_ responses of stomatal conductance in both gymnosperm and angiosperm trees. New Phytol 237:1229–1241. 10.1111/nph.18618 36373000 10.1111/nph.18618

[CR22] Gielen B, Liberloo M, Bogaert J, Calfapietra C, De Angalis P, Miglietta F, Scarascia-Mugnozza G, Ceulemans R (2003) Three years of free-air CO_2_ enrichment (POPFACE) only slightly affect profiles of light and leaf characteristics in closed canopies of *Populus*. Glob Change Biol 9:1022–1037. 10.1046/j.1365-2486.2003.00644.x

[CR23] Gorman MJ (2023) Iron homeostasis in insects. Annu Rev Entomol 68:51–67. 10.1146/annurev-ento-040622-092836 36170642 10.1146/annurev-ento-040622-092836 PMC10829936

[CR24] Günthardt-Goerg MS, Vollenweider P (2015) Responses of beech and spruce foliage to elevated carbon dioxide, increased nitrogen deposition and soil type. AoB Plants 7:plv067. 10.1093/aobpla/plv067 26092041 10.1093/aobpla/plv067 PMC4522038

[CR25] Guo Z, Zhuang M, Yang L, Li Y, Wu S, Chen S (2021) Differentiated mineral nutrient management in two bamboo species under elevated CO_2_ environment. J Environ Manag 279:111600. 10.1016/j.jenvman.2020.111600 10.1016/j.jenvman.2020.111600 33160742

[CR26] Hänsch R, Mendel RR (2009) Physiological functions of mineral micronutrients (Cu, Zn, Mn, Fe, Ni, Mo, B, Cl). Curr Opin Plant Biol 12:259–266. 10.1016/j.pbi.2009.05.006 19524482 10.1016/j.pbi.2009.05.006

[CR27] Hart KM, Curioni G, Blaen P, Harper NJ, Miles P, Lewin KF, Nagy J, Bannister EJ, Cai XM, Thomas RM, Krause S, Tausz M, MacKenzie AR (2020) Characteristics of free air carbon dioxide enrichment of a northern temperate mature forest. Glob Change Biol 26:1023–1037. 10.1111/gcb.14786 10.1111/gcb.14786 PMC7027798 31376229

[CR28] Hasanuzzaman M, Bhuyan M, Nahar K, Hossain M, Mahmud J, Hossen M, Masud A, Moumita FM (2018) Potassium: a vital regulator of plant responses and tolerance to abiotic stresses. Agronomy 8:31. 10.3390/agronomy8030031

[CR29] Hrdina A, Iatsenko I (2022) The roles of metals in insect–microbe interactions and immunity. Curr Opin Insect Sci 49:71–77. 10.1016/j.cois.2021.12.004 34952239 10.1016/j.cois.2021.12.004

[CR30] Inoue A, Takasaki H (2016) Seasonal change and diversity of pollen-source plants used by the Japanese honeybee (*Apis cerana japonica*): a lowland case in Southwestern Honshu. Naturalistae 20:47–56

[CR31] Isanta-Navarro J, Prater C, Peoples LM, Loladze I, Phan T, Jeyasingh PD, Church MJ, Kuang Y, Elser JJ (2022) Revisiting the growth rate hypothesis: towards a holistic stoichiometric understanding of growth. Ecol Lett 25:2324–2339. 10.1111/ele.14096 36089849 10.1111/ele.14096 PMC9595043

[CR32] Jamieson MA, Burkle LA, Manson JS, Runyon JB, Trowbridge AM, Zientek J (2017) Global change effects on plant–insect interactions: the role of phytochemistry. Curr Opin Insect Sci 23:70–80. 10.1016/j.cois.2017.07.009 29129286 10.1016/j.cois.2017.07.009

[CR33] Jay SC (1964) The Cocoon of the Honey Bee, *Apis mellifera* L. Can Entomol 96:784–792. 10.4039/Ent96784-5

[CR34] Kaspari M (2020) The seventh macronutrient: how sodium shortfall ramifies through populations, food webs and ecosystems. Ecol Lett 23:1153–1168. 10.1111/ele.13517 32380580 10.1111/ele.13517

[CR35] Kaspari M (2021) The invisible hand of the periodic table: how micronutrients shape ecology. Annu Rev Ecol Evol Syst 52:199–219. 10.1146/annurev-ecolsys-012021-090118

[CR36] Kaspari M, Welti EAR (2024) Nutrient dilution and the future of herbivore populations. Trends Ecol Evol. 10.1016/j.tree.2024.05.001 38876933 10.1016/j.tree.2024.05.001

[CR37] Lau P, Bryant V, Rangel J (2018) Determining the minimum number of pollen grains needed for accurate honey bee (*Apis mellifera*) colony pollen pellet analysis. Palynology 42:36–42. 10.1080/01916122.2017.1306810

[CR38] Lau P, Lesne P, Grebenok RJ, Rangel J, Behmer ST (2022) Assessing pollen nutrient content: a unifying approach for the study of bee nutritional ecology. Philos Trans R Soc B Biol Sci 377:20210510. 10.1098/rstb.2021.0510 10.1098/rstb.2021.0510 PMC9058549 35491590

[CR39] Le Thiec D, Dixon M, Loosveldt P, Garrec JP (1995) Seasonal and annual variations of phosphorus, calcium, potassium and manganese contents in different cross-sections of *Picea abies* (L.) Karst. needles and *Quercus rubra* L leaves exposed to elevated CO_2_. Trees 10:55–62. 10.1007/bf00192184

[CR40] Lill R, Mühlenhoff U (2006) Iron-Sulfur Protein Biogenesis in Eukaryotes: Components and Mechanisms. Annu Rev Cell Dev Biol 22:457–486. 10.1146/annurev.cellbio.22.010305.104538 16824008 10.1146/annurev.cellbio.22.010305.104538

[CR41] Loladze I (2014) Hidden shift of the ionome of plants exposed to elevated CO_2_ depletes minerals at the base of human nutrition. eLife 3:e02245. 10.7554/elife.02245 24867639 10.7554/eLife.02245 PMC4034684

[CR42] MacKenzie AR, Krause S, Hart KM et al (2021) BIFoR FACE: Water–soil–vegetation–atmosphere data from a temperate deciduous forest catchment, including under elevated CO_2_. Hydrol Process. 10.1002/hyp.14096

[CR43] Martins NP, Fuchslueger L, Fleischer K et al (2021) Fine roots stimulate nutrient release during early stages of leaf litter decomposition in a Central Amazon rainforest. Plant Soil 469:287–303. 10.1007/s11104-021-05148-9

[CR44] Mayoral C, Ioni S, Luna E, Crowley LM, Hayward SA, Sadler JP, MacKenzie AR (2023) Elevated CO_2_ does not improve seedling performance in a naturally regenerated oak woodland exposed to biotic stressors. Front Forests Glob Change 6:1278409. 10.3389/ffgc.2023.1278409

[CR45] Morrissey J, Guerinot ML (2009) Iron uptake and transport in plants: the good, the bad, and the ionome. Chem Rev 109:4553–4567. 10.1021/cr900112r 19754138 10.1021/cr900112r PMC2764373

[CR46] Norby RJ, De Kauwe MG, Domingues TF, Duursma RA, Ellsworth DS, Goll DS, Lapola DM, Luus KA, MacKenzie AR, Medlyn BE, Pavlick R, Rammig A, Smith B, Thomas R, Thonicke K, Walker AP, Yang X, Zaehle S (2016) Model–data synthesis for the next generation of forest free-air CO_2_ enrichment (FACE) experiments. New Phytol 209:17–28. 10.1111/nph.13593 26249015 10.1111/nph.13593

[CR47] Oksanen L (2001) Logic of experiments in ecology: is pseudoreplication a pseudoissue? Oikos 94:27–38. 10.1034/j.1600-0706.2001.11311.x

[CR48] Oksanen J, Simpson G, Blanchet F, Kindt R, Legendre P, Minchin P, O’Hara R, Solymos P, Stevens M, Szoecs E, Wagner H, Barbour M, Bedward M, Bolker B, Borcard D, Carvalho G, Chirico M, De Caceres M, Durand S, Evangelista H, FitzJohn R, Friendly M, Furneaux B, Hannigan G, Hill M, Lahti L, McGlinn D, Ouellette M, Ribeiro Cunha E, Smith T, Stier A, Ter Braak C, Weedon J (2022) _vegan: Community Ecology Package_. R package version 2.6–4. https://CRAN.R-project.org/package=vegan.

[CR49] Ollerton J (2021) Pollinators and pollination: nature and society. Pelagic Publishing Limited, London

[CR50] Peñuelas J, Fernández-Martínez M, Vallicrosa H, Maspons J, Zuccarini P, Carnicer J, Sanders TGM, Krüger I, Obersteiner M, Janssens IA, Ciais P, Sardans J (2020) Increasing atmospheric CO_2_ concentrations correlate with declining nutritional status of European forests. Commun Biol 3:125. 10.1038/s42003-020-0839-y 32170162 10.1038/s42003-020-0839-y PMC7070084

[CR51] Peterson RA (2021) Finding Optimal Normalizing Transformations via bestNormalize. R J 13(1):310–329. 10.32614/RJ-2021-041

[CR52] Pinheiro J, Bates D, R Core Team (2023) _nlme: linear and nonlinear mixed effects models_. R Package version 3.1–162. https://CRAN.R-project.org/package=nlme

[CR53] Quantum GIS Development Team (2023) QGIS geographic information system. Open Source Geospatial Foundation Project. https://qgis.org/pl/site/index.html. Accessed Sep 1 2023

[CR54] R Core Team (2023) R: a language and environment for statistical computing. R Foundation for Statistical Computing, Vienna, Austria

[CR55] Rennenberg H, Dannenmann M (2015) Nitrogen nutrition of trees in temperate forests—the significance of nitrogen availability in the pedosphere and atmosphere. Forests 6:2820–2835. 10.3390/f6082820

[CR56] Robinson EA, Ryan GD, Newman JA (2012) A meta-analytical review of the effects of elevated CO_2_ on plant-arthropod interactions highlights the importance of interacting environmental and biological variables. New Phytol 194:321–336. 10.1111/j.1469-8137.2012.04074.x 22380757 10.1111/j.1469-8137.2012.04074.x

[CR57] Sardans J, Peñuelas J (2015) Potassium: a neglected nutrient in global change. Glob Ecol Biogeogr 24:261–275. 10.1111/geb.12259

[CR58] Sgouridis F, Reay M, Cotchim S, Ma J, Radu A, Ullah S (2023) Stimulation of soil gross nitrogen transformations and nitrous oxide emission under free air CO_2_ enrichment in a mature temperate oak forest at BIFoR-FACE. Soil Biol Biochem 184:109072. 10.1016/j.soilbio.2023.109072

[CR59] Splitt A, Skórka P, Strachecka A, Borański M, Teper D (2021) Keep trees for bees: pollen collection by *Osmia bicornis* along the urbanization gradient. Urban for Urban Green 64:127250. 10.1016/j.ufug.2021.127250

[CR60] Splitt A, Schulz M, Skórka P (2022) Current state of knowledge on the biology and breeding of the solitary bee – *Osmia bicornis*. J Apic Res 61:163–179. 10.1080/00218839.2021.1957610

[CR61] Sterner R, Elser J (2002) Ecological stoichiometry: the biology of elements from molecules to the biosphere. Princeton University Press, Princeton

[CR62] Ordnance Survey (2023) Open Data website. OS VectorMap® District. https://osdatahub.os.uk/downloads/open/VectorMapDistrict. Accessed Sep 1 2023

[CR63] Tylianakis JM, Didham RK, Bascompte J, Wardle DA (2008) Global change and species interactions in terrestrial ecosystems. Ecol Lett 11:1351–1363. 10.1111/j.1461-0248.2008.01250.x 19062363 10.1111/j.1461-0248.2008.01250.x

[CR64] Welti EAR, Kaspari M (2021) Sodium addition increases leaf herbivory and fungal damage across four grasslands. Funct Ecol 35:1212–1221. 10.1111/1365-2435.13796

[CR65] Welti EAR, Roeder KA, de Beurs KM, Joern A, Kaspari M (2020) Nutrient dilution and climate cycles underlie declines in a dominant insect herbivore. Proc Natl Acad Sci USA 117:7271–7275. 10.1073/pnas.1920012117 32152101 10.1073/pnas.1920012117 PMC7132292

[CR66] Wilson RJ, Fox R (2021) Insect responses to global change offer signposts for biodiversity and conservation. Ecol Entomol 46:699–717. 10.1111/een.12970

[CR67] Ziska LH, Pettis JS, Edwards J, Hancock JE, Tomecek MB, Clark A, Dukes JS, Loladze I, Polley HW (2016) Rising atmospheric CO_2_ is reducing the protein concentration of a floral pollen source essential for North American bees. Proc R Soc B Biol Sci 283:20160414. 10.1098/rspb.2016.0414 10.1098/rspb.2016.0414 PMC4843664 27075256

