# Supplementary material for: Elevated atmospheric CO2 alters the multi-element stoichiometry of pollen-bearing oak flowers, with possible negative effects on bees
Source: Oecologia. 2024 Sep 8;206(1-2):101–14. doi: 10.1007/s00442-024-05610-2 (PMC11489284; doi:10.1007/s00442-024-05610-2)
Supplement: Supplementary file 1 — Supplementary file1 (DOCX 581 KB) [file 442_2024_5610_MOESM1_ESM.docx]

**Elevated atmospheric CO_2_ alters the multi-element stoichiometry of pollen-bearing oak flowers, with possible negative effects on bees**

Zuzanna M. Filipiak^1*^, Carolina Mayoral^2,3^, Sophie A. Mills^2,4^, Scott A. L. Hayward^2,3^ and Sami Ullah^2,4^

^1^Institute of Environmental Sciences, Jagiellonian University, Gronostajowa 7, 30-387 Kraków, Poland

^2^Birmingham Institute of Forest Research, University of Birmingham, Edgbaston B15 2TT, England

^3^School of Biosciences, University of Birmingham, Birmingham, United Kingdom, B15 2TT

^4^School of Geography, Earth and Environmental Sciences, University of Birmingham, Edgbaston, B15 2TT, UK

^*^**Corresponding author**: Zuzanna M. Filipiak

Email: zuzanna.filipiak@uj.edu.pl

Phone: (+48)126646878

**Supplementary Tables**

**Table S1.** The concentrations (mean ± standard deviation) of 12 elements present in the pollen-bearing flowers obtained from pedunculate oak (*Quercus robur*), collected from ambient (aCO_2_) and elevated (eCO_2_) treatments at the Birmingham Institute of Forest Research (BIFoR) Free Air Carbon Enrichment (FACE) facility.

| **Treatment** | **C (%)** | **N (%)** | **P (%)** | **S (%)** | **K (mg/kg)** | **Na (mg/kg)** | **Ca (mg/kg)** | **Mg (mg/kg)** | **Cu (mg/kg)** | **Zn (mg/kg)** | **Fe (mg/kg)** | **Mn (mg/kg)** |
| --- | --- | --- | --- | --- | --- | --- | --- | --- | --- | --- | --- | --- |
| **aCO_2_** | 49.6 ± 1.02 | 4.7 ± 0.19 | 0.3 ± 0.08 | 0.2 ± 0.02 | 19185.6 ± 1274.07 | 323.9 ± 76.74 | 3613.7 ± 915.67 | 3833.5 ± 288.36 | 22.8 ± 2.18 | 67.0 ± 3.87 | 117.2 ± 9.16 | 555.9 ± 149.05 |
| **eCO_2_** | 49.8 ± 0.51 | 4.8 ± 0.70 | 0.4 ± 0.22 | 0.2 ± 0.02 | 17626.8 ± 1012.82 | 291.1 ± 109.10 | 3890.7 ± 1338.44 | 4090.0 ± 309.38 | 21.4 ± 3.67 | 65.0 ± 8.35 | 98.5 ± 15.46 | 507.2 ± 229.66 |

**Supplementary Figures**


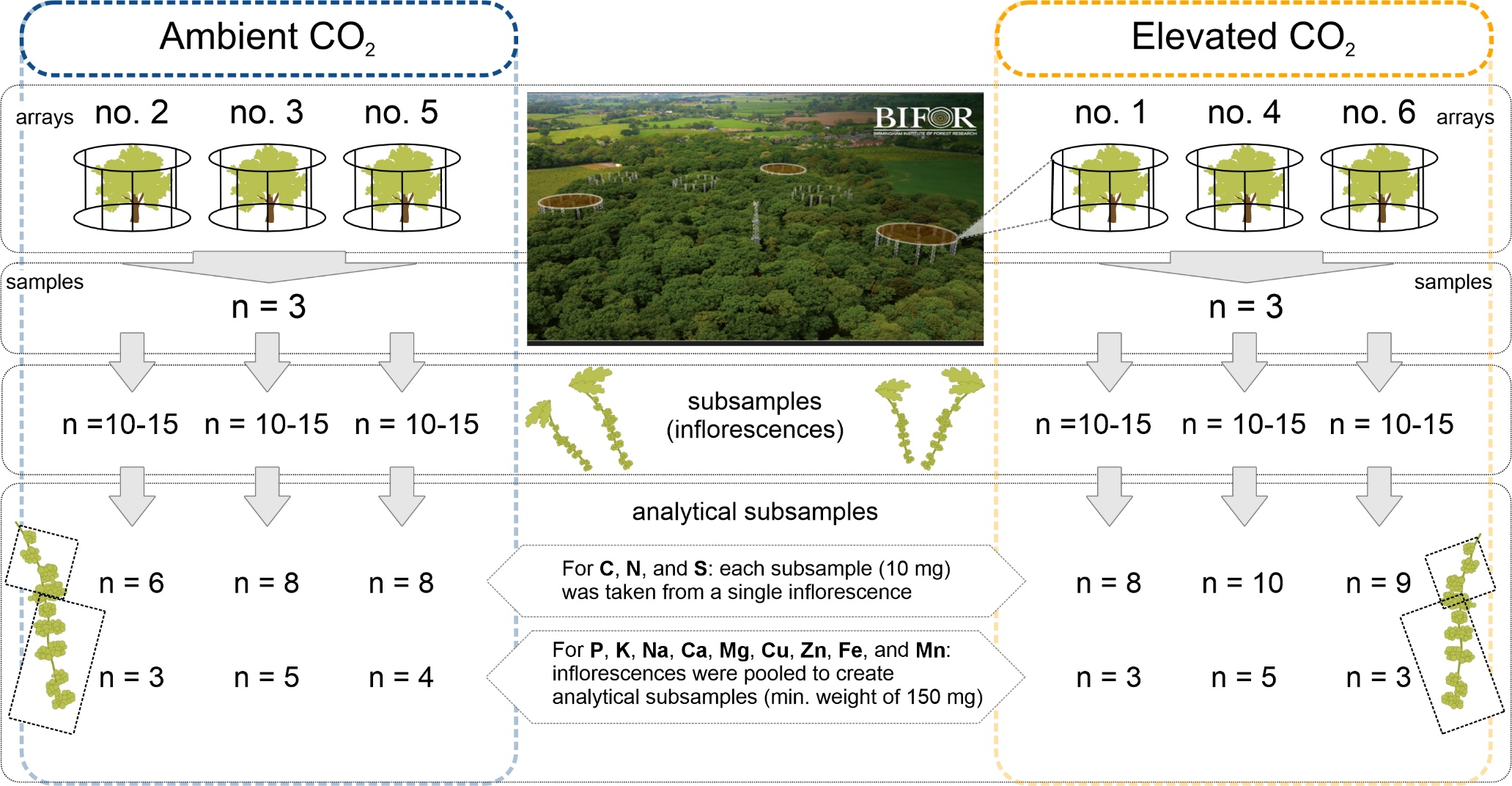


**Fig. S1.** Material collection from the Birmingham Institute of Forest Research (BIFoR) Free Air Carbon Enrichment (FACE) facility. The inserted aerial photograph shows the FACE arrays, with fumigated arrays highlighted in red (photo used with permission of S. Ullah). Samples were collected from ambient CO_2_ (aCO_2_) (left panel) and elevated CO_2_ (eCO_2_) (right panel) treatments. One oak tree was assessed per array, resulting in one true replicate per array. From each tree, 10–15 inflorescences were collected, with pollen-bearing flowers used to create analytical samples. The number of analytical samples for C, N, S, and other elements (P, K, Na, Ca, Mg, Cu, Zn, Fe, Mn) varies due to the minimum sample size required for analyses.
